# Melting Profile of DNA in Crowded Solution: Model-Based Study

**DOI:** 10.3390/ijms26115305

**Published:** 2025-05-31

**Authors:** Neha Mathur, Amar Singh, Navin Singh

**Affiliations:** 1 Department of Physics, Birla Institute of Technology & Science, Pilani 333031, Indianavin@pilani.bits-pilani.ac.in (N.S.); 2 Center for Computational Biology, The University of Kansas, Lawrence, KS 66047, USA

**Keywords:** dsDNA, DNA melting, molecular crowding, thermal ensemble

## Abstract

Recent advances in molecular dynamics (MD) simulations and the introduction of artificial intelligence (AI) have resulted in a significant increase in accuracy for structure prediction. However, the cell is a highly crowded environment consisting of various macromolecules, such as proteins and nucleic acids. The macromolecular crowding and solution conditions, such as temperature, ion concentration, and the presence of crowders, significantly influence the molecular interactions between and structural changes in proteins and nucleic acids. In this study, we investigate the presence of crowders and their effect on the melting of DNA molecules by analyzing melting profiles of short and long heterogeneous DNA duplexes. In particular, we examine how multiple inert crowders, randomly distributed along long DNA chains, influence DNA melting. We find that the presence of crowders stabilizes double-stranded DNA (dsDNA), with this effect being more pronounced in short DNA duplexes. These findings complement in vitro observations and improve our understanding of dsDNA in cell-like environments.

## 1. Introduction

The presence of various biomolecules, such as proteins, nucleic acids, saccharides, lipids, and metabolites, makes the cellular environment highly crowded [1,2]. These molecules are known to occupy about 30–40% of the space within a cell [3]. In recent years, researchers have been investigating the effect of crowders and different solution conditions on various biological activities of macromolecules in the cell [4]. For example, researchers have studied protein stability [5], protein–protein interactions [6], protein diffusion [7], and the structures and functions of nucleic acids [8,9] in crowded environments. Crowders also affect DNA and its condensation into the nucleoid of bacterial cells [10], DNA polymerase reactions [11], DNA melting [12,13], and unzipping [14,15]. These studies elucidate numerous aspects of biomolecular activities and interactions under the influence of molecular crowders. The crowding impact is multifaceted and can vary depending on factors such as crowder size, concentration, and the specific molecules involved. For example, the differential stability of DNA in a wide range of salt concentrations [16,17] or a crowding of specific molecules can stabilize or destabilize DNA depending on their charge and binding properties [18]. However, a detailed understanding of how the spatial distribution and strength of molecular crowders affect the melting behavior of heterogeneous DNA sequences remains limited. In this study, we investigate the effect of crowders’ presence on the melting of DNA molecules by analyzing melting profiles of short and long DNA chains. The melting profile is compared with the experimental melting curve [19] of short DNA duplexes to identify likely crowder locations near DNA molecules. Additionally, the melting temperature is determined for all possible unique distributions of crowders.

To investigate DNA melting from a theoretical perspective, a range of classical and statistical mechanical models have been developed to describe DNA conformational dynamics. Soon after the discovery of the DNA double-helix structure, attempts were made to study and model DNA denaturation, as well as to use classical models such as worm-like chain (WLC) [20,21] and freely jointed chain (FJC) [22] to study conformational and mechanical properties. Among these, the statistical mechanical Poland–Scheraga [23] and Peyrard–Bishop [24] models are better suited for studying the sequence-dependent melting behavior and bubble formation in DNA. Figure S1 shows these models are still popular and are being used to study DNA dynamics and base-pair fluctuations. In recent years, there has been significant improvement in generating and analyzing large data-sets of DNA melting curves either using high-throughput experimental methods or improved computational models [25,26,27]. Also, molecular dynamics (MD) simulations provide a detailed, atomistic view of DNA, which allows scientists to observe structural changes in DNA [28,29]. By simulating every atom in the DNA molecule and its surrounding environment, MD captures the precise interactions at the base-pair level. Despite the differences in different approaches to modelling DNA and its structural changes, these methods aim to uncover how DNA behaves under various conditions. The MD simulations can be customized to focus on particular atomic interactions by tweaking simulation parameters or force field information. On the other hand, Hamiltonian-based theoretical models, such as the Peyrard–Bishop–Dauxois (PBD) [30] model, can be modified to include interactions characteristic within certain environments, such as the effect of molecular crowders or salts, etc. The PBD model simplifies DNA into a one-dimensional chain of base-pairs, concentrating primarily on the interactions between these pairs. This model does not account for every atom but instead focuses on how the base-pairs interact [8]. By reducing the atomic complexity, the PBD model manages larger DNA systems over extended periods with less computational demand [31]. This approach is particularly useful for examining phenomena like DNA denaturation and the impact of mechanical forces on DNA structure [32]. Consequently, the PBD model excels at exploring broad DNA behavior patterns and responses under various conditions, making it a powerful tool for large-scale studies [33,34]. In the present work, we study the melting of DNA molecules in the presence of molecular crowders in a thermal ensemble using the PBD model. With appropriate modifications to the PBD model for crowded base-pairs, we provide a clear assessment of how crowders affect the melting of DNA duplexes.

## 2. Results and Discussion

### 2.1. Melting of Short DNA Duplex

To investigate the melting of short DNA chains, we adopted DNA sequences from the study by Ghosh et al. [19], in which ultraviolet (UV) melting measurements were performed in crowded solutions containing polyethylene glycol (PEG). The reported absorbance vs. temperature curve reflects the fraction of open base-pairs, ϕ(T), and serves as the experimental melting profile. In our model, we compute ϕ(T) as the average fraction of disrupted base-pairs using the PBD framework, allowing for direct comparison with the experimental data. The following sequences were examined in this study: $5^{\prime }$-GGGAGAAG-$3^{\prime }$ (referred to as chain-A) and $5^{\prime }$-GGAAGAGG-$3^{\prime }$ (referred to as chain-B). Please note that the letters representing the sequences are assigned as follows: “*G*” stands for GC or CG base-pairs, while “*A*” stands for AT or TA base-pairs. These chains represent two separate duplexes, each forming a DNA duplex with its respective complementary strand. The selected sequences vary in GC content and distribution, making them representative examples to test our modified PBD model’s ability to reproduce experimental melting behavior under crowded conditions.

To incorporate crowders into the theoretical model, their presence is typically represented by modifying the model Hamiltonian, rather than explicitly simulating them [35,36,37]. Since the dissociation of base-pairs is represented by Morse potential, increasing the potential depth is a way to simulate the stabilization effect that crowders might have on the DNA. This adjustment reflects the increased environmental pressure and reduced volume available to the DNA due to the presence of crowding agents. A base-pair that is surrounded by a crowder needs very high energy to break the hydrogen bond. In the PBD model, the base-pairs can move only along the *y* direction; thus, the space available to the base-pair surrounded by a crowder will be restricted. The crowders are the biomolecules that can move due to the thermal fluctuation in the DNA molecule. For the zeroth-order approximation, we assume that a base-pair, which is surrounded by a crowder, requires a very high amount of energy to overcome the potential barrier or to break the hydrogen bonds. Since the bond breaking in the PBD model is reflected through the barrier height of the Morse potential, we increase the value of $D_{0}$ by a scaling factor α for the pair surrounded by a crowder. Based on solution conditions, such scaling of the base-pair interaction potential has been previously implemented to study DNA melting in the presence of salt [37,38,39,40], crowders [13], or solvent molecules [15], and the charge transport in a DNA model with solvent interaction [41]. In these studies, the values of α range from 1 to 1.5. We modify the depth of potential as $D=\alpha *D_{0}$, where α=1.5 is for the crowded site and represents the optimal value for reproducing the experimental melting curve.

**Figure 1:**
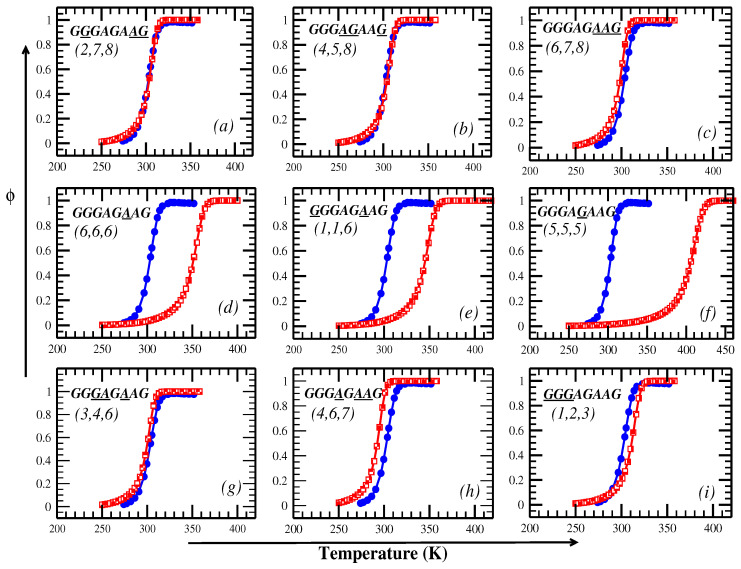
The change in the fraction of open base-pairs with temperature for chain-A (GGGAGAAG) for different locations of the crowders (shown within parentheses in sub figures (**a**–**i**)) around base-pairs (underlined letters in sequence). The numerical results obtained from the PBD model are shown in red, while blue corresponds to the experimental melting curve [19].

In line with the experiments, where 40% of the volume is occupied by crowders, we assume that 40% of the base-pairs are crowded; therefore, three randomly selected base-pairs are crowded. The crowders, typically inert molecules that mimic the crowded cellular environment, are slowly diffusive and restrict base-pair fluctuations. We consider different locations of these crowders and investigate the effect of crowders and their locations on the melting profile of DNA molecules. To provide a visual comparison with ultraviolet (UV) melting curves from other experiments, the fraction of open base-pairs (ϕ) as a function of temperature is shown in Figure 1. For a 40% crowded sequence, i.e., three base-pairs, there can be many distributions of crowders; we present the results for nine different distributions in Figure 1. The melting curves in the absence of crowders and with more than three crowders are presented in Figure S3. The melting curve is influenced by the positioning of the crowders, as evident from Figure 1a–i. In Figure 1a, the crowders are present at the 2nd,7th, and 8th sites; this restricts the chain to opening from the ends and forces it to open in the mid region. Similarly, we place the crowders at other locations to find the melting curve matches with the experiments. In the experiments, the crowder’s location around the DNA was not static; hence, we also explored the possibility of one site having more than one crowder—see Figure 1d–f. For such cases, we observe higher melting temperatures, indicating that in the experiment the crowders are distributed throughout the chain. Among various possible distributions, the crowder’s distribution as (2,7,8) or (4,5,8) exhibits the closest match to the experimental melting curve. The crowders might be present in such a way that two crowders are located in one half of the chain while the third crowder is present at the other end of the chain.

**Figure 2:**
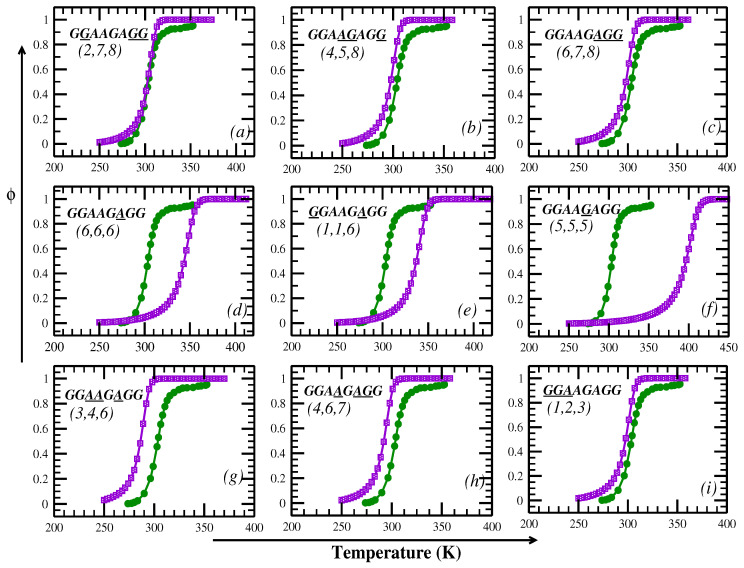
The change in the fraction of open base-pairs with temperature for chain-B (GGAAGAGG) for different locations of the crowders (shown within parentheses in sub figures (**a**–**i**)) around base-pairs (underlined letters in sequence). The numerical results obtained from the PBD model are shown in purple while green corresponds to the experimental melting curve [19].

**Figure 3:**
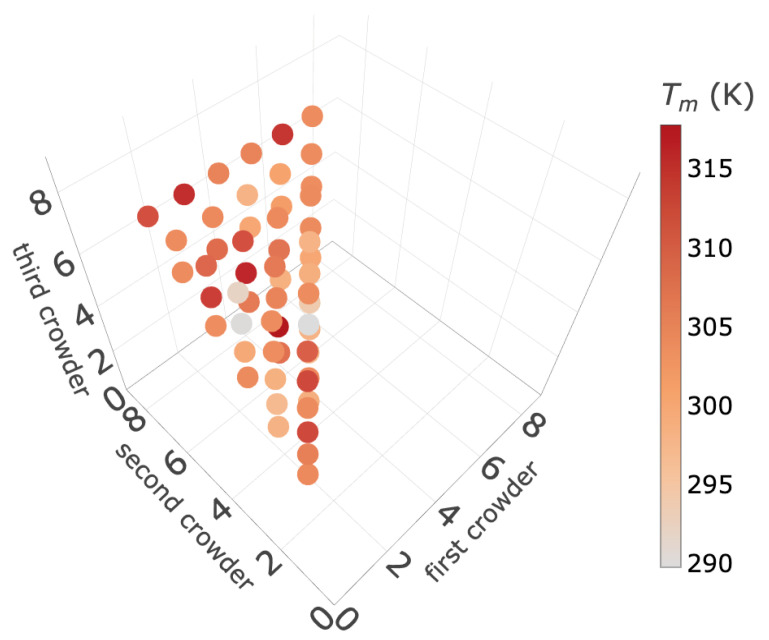
Melting temperature ($T_{m}$) for different locations of three crowders along DNA chain is shown for 56 unique, non-redundant distributions.

To check whether the results are sensitive to the sequence, we consider another sequence (chain-B) for which the experimental results are available [19]. The melting curves for different distributions of crowders in comparison with the experimental curve are presented in Figure 2. It is clear from these results that when the crowders are at (2,7,8), there is a close match with the experimental melting curve, demonstrating that the location of crowders is very important and the melting profile is very sensitive not only to the sequence but also to the location of crowders. For chain-B, we do not have a good match for the distributions (4,5,8) for which we obtained a good match for chain-A. It is important to note that both the chains have five GC and three AT pairs and the crowded sites have the same kind of pair (AGG). However, there is a difference in the sequence of the base-pairs in the other half of the chain where there is no crowder. Chain-A has $5^{\prime }-GGGA$ pairs while chain-B has $5^{\prime }-GGAA$ pairs where there is no crowder. This introduces a difference in the entropic contribution from pairs of varying H-bond strengths and their resulting stacking interactions. Consequently, the same crowder distribution (4, 5, 8), which provides a good match for chain-A, does not yield similar agreement for chain-B. This highlights that the melting behavior is highly sensitive not only to crowder location but also to the sequence context outside the crowded region. Also, the overall sequence composition of chain-A contains five GC base-pairs, including strong terminal GC pairs, while chain-B has only four GC base-pairs and more evenly distributed AT pairs. This composition difference likely contributes to the higher stability (and melting temperature) observed for chain-A in both experimental and model-based melting curves. To explore the impact of crowders and their specific locations on the DNA melting profile, we randomly positioned the crowders. For a chain of eight base-pairs with three crowders, there are 120 possible crowder distributions. Since the crowders are identical, there are only 56 unique distributions. The melting temperatures (denoted as $T_{m}$) for these 56 configurations are shown in Figure 3 and the actual values are given in Table S1. The average value of $T_{m}$ across all 56 configurations is found to be 303.16 K, which closely aligns with the experimental finding of approximately 303.5 K.

### 2.2. Melting of Long DNA Duplex

**Figure 4:**
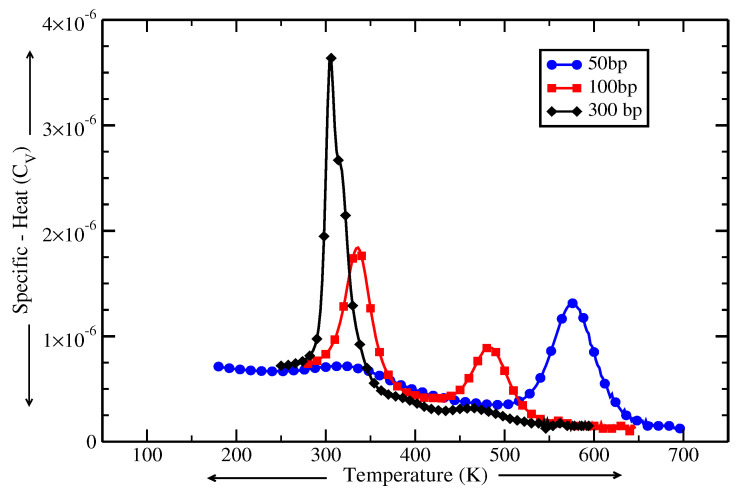
The variation in specific heat ($C_{v}$) with temperature for 50-, 100-, and 300-base-pair DNA chains in the presence of five crowders.

In biological systems, the distribution of crowders and their strengths are diverse and random inside the cell. In order to replicate a cell-like environment, we consider long DNA chains and a diverse array of crowders, each with unique strengths distributed in a random and heterogeneous manner. We selected five crowders and assigned different values of α to each crowder, specifically 5, 6, 7, 8, and 9, keeping other model parameters the same as for short chains. We investigate three DNA chain lengths, 50, 100, and 300 base-pairs, and crowder locations are randomly assigned along the chain. For example, in the 50-base-pair chain, the crowders are located at positions 33, 36, 27, 15, and 43 and each crowder has a different intensity based on its corresponding scaling factor α—5, 6, 7, 8, and 9 respectively. Similarly, for the 100- and 300-base-pair chains, the crowder locations are 83, 86, 77, 15, and 93 & 283, 286, 177, 115, and 293, respectively. This random distribution of crowders, exerting different degrees of confinement and stabilization on DNA, represents the heterogeneity of biological systems. We calculate the specific heat, $C_{v}$ (see methods), of these chains with crowded base-pairs, and the results are shown in Figure 4. Interestingly, we observe that the number of peaks and their locations (representing a local melting) are different in all three chains. In the 50-base-pair chain, the first peak is of lower height than the second peak. In contrast, for the 100-base-pair chain, the second peak is of lower height. Surprisingly, for the 300-base-pair chain, the second peak nearly disappears. These results indicate that the presence of crowders in the cell affects the melting behavior of DNA molecules of different lengths in unique ways. Additionally, the area under the curve of the specific heat, which is related to the system’s entropy, differs for each chain length. The melting of the 50-base-pair chain is found to be less entropic compared to the others, while the 300-base-pair chain exhibits high conformational entropy. This variation in entropic contribution also stems from the differing relative fractions of crowded sites, which are approximately 10%, 5%, and 1.7% for the 50-, 100-, and 300-base-pair chains, respectively. The increase in entropy drives the system into a disordered state and the 300-base-pair chain has the lowest melting temperature. In our earlier study, we showed the any restriction to the base-pair fluctuations leads to a higher $T_{m}$ below the thermodynamic limit for chain length, which was found to be 600 base-pairs [32,42].

**Figure 5:**
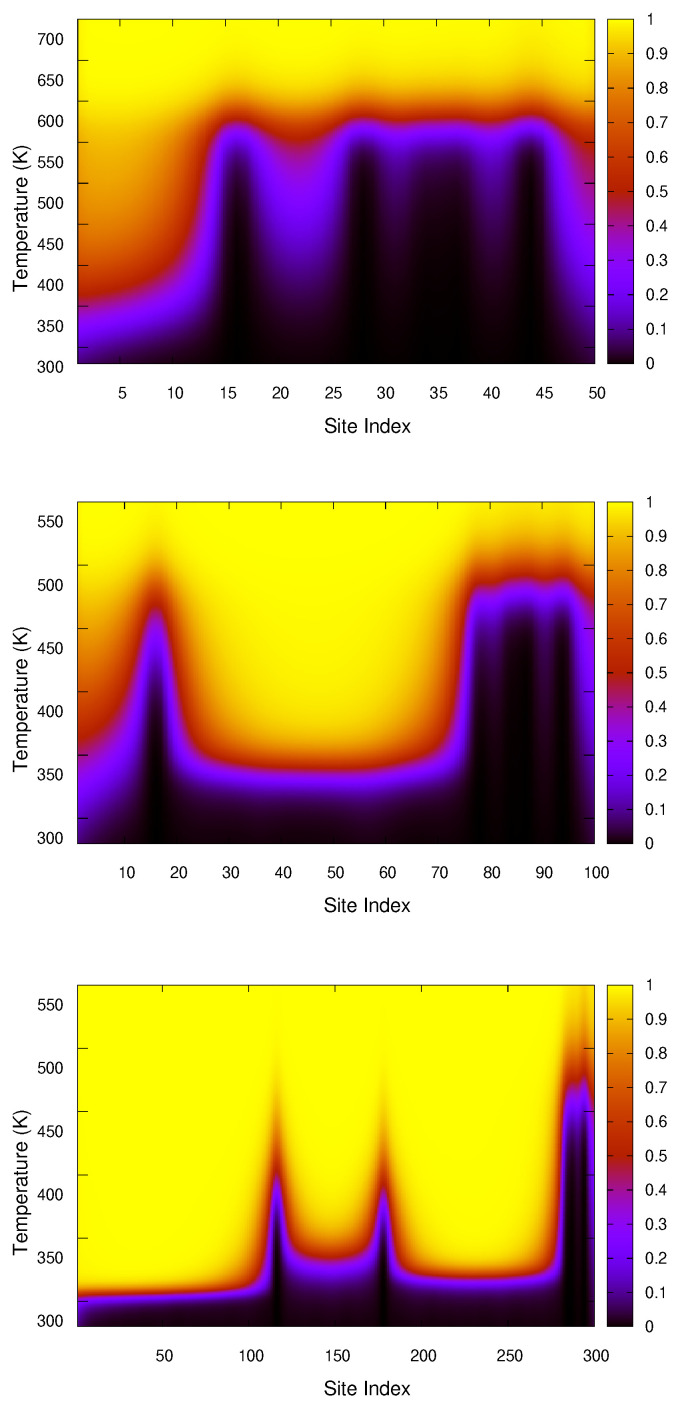
The opening probabilities of 50-, 100-, and 300-base-pair long DNA duplexes in the presence of five crowders. The location as well as the strength of the crowders are visible through the peaks in the black region.

We further calculated the opening probabilities for these chains to gain a deeper understanding of the opening process. The probability of opening of the $i^{th}$ pair is defined as follows:

(1) $$P_{i}=\frac{1}{Z_{c}}\int_{y_{0}}^{\infty }dy_{n}\exp\left[-\beta H(y_{i},y_{i+1})\right]Z_{i}$$

(2) $$Z_{i}=\frac{1}{h}\int_{-\infty }^{\infty }\prod_{\begin{array}{c} j=1\\ j\neq i \end{array}}^{N}dy_{j}\exp\left[-\beta H(y_{j},y_{j+1})\right]$$

where $Z_{c}$ is the configurational part of the partition function and $y_{0}$ represents the distance threshold for DNA base-pair separation. Studies have shown that a fluctuation cut-off of about 1.5–2.2 Å yields results consistent with DNA melting experiments and the physical chemistry of base-pair disruption [43,44]. Figure S2 shows the average base-pair separation, with 〈y〉 as a function of *T*. We observe a divergence in 〈y〉 around 2 Å, which serves as a practical and computationally meaningful criterion for distinguishing between closed and open states within the model. Therefore, we choose $y_{0}$ = 2.0 Å and keep the rest of the model parameters the same. The opening probabilities are presented in Figure 5, providing insights into the base-pair opening characteristics of each chain.

As discussed above, for the 50-base-pair chain, we observe that initially only a few base-pairs (about 10) open, which explains the smaller peak in the plot. The majority of the chain opens at higher temperatures but within a narrow range, resulting in a substantial increase in the number of open base-pairs, corresponding to a higher peak in specific heat. In the case of the 100-base-pair chain, a significant bubble forms for base-pairs 20–70, where roughly 50% of the chain has no crowders in the region and are in the open state. However, the ends of the chain remain intact due to the presence of a crowder, which requires more energy to open the bonds. This disparity in energy requirements is the reason why the first peak in this case is higher than the second one. In the 300-base-pair chain, we observe a large bubble spanning base-pairs 200–275, as well as a larger opening at one end (base-pairs 1–95). Additionally, there is a smaller bubble between base-pairs 125–175 in the middle section of the chain. These factors contribute to the higher entropy of the 300-base-pair chain. Another important aspect contributing to its increased entropy is the reduction in the fraction of crowded sites, which is approximately ∼1.7% for this chain. In comparison, the 50-base-pair chain has 10% crowded sites, and the 100-base-pair chain has 5% crowded sites.

## 3. Materials and Methods

To study the effect of molecular crowders on the melting of DNA molecules in a thermal ensemble, we use the well-known Peyrard–Bishop–Dauxois model (PBD) [24,30]. The model is quasi-one-dimensional and expresses the dynamics of the molecule through the stretching of the hydrogen bonds. The interactions in the DNA, containing *N* base-pairs, are represented as

(3) $$H=\sum_{n=1}^{N}\left[\frac{p_{n}^{2}}{2m}+V_{m}\left(y_{n}\right)\right]+\sum_{n=1}^{N-1}\left[V_{s}\left(y_{n},y_{n-1}\right)\right]$$

where $y_{n}$ represents the separation between two bases in a pair. The separation $y_{n}=0.0$ Å refers to the equilibrium position of two bases in a pair. The first term of the model is the momentum term, which is $p_{n}=m{\dot{y}}_{n}$. We use the same reduced mass, m=300 amu, for both the AT and GC base-pairs. The interaction between the nearest base-pairs along the chain—the stacking interaction—is represented by

(4) $$V_{s}\left(y_{n},y_{n-1}\right)=\frac{\kappa }{2}{\left(y_{n}-y_{n-1}\right)}^{2}\left[1+\rho e^{-b(y_{n}+y_{n-1})}\right]$$

The single-strand elasticity is represented by κ, the anharmonicity in strand elasticity is represented by ρ, and parameter *b* describes the range of anharmonicity. The values of *k* and ρ define the sharpness in the transition from double-strand to single-strand [35,45,46]. The Morse potential represents the hydrogen bond between the two bases in the $n^{\mathrm{th}}$ pair.

(5) $$V_{M}\left(y_{n}\right)=D_{n}{\left(e^{-a_{n}y_{n}}-1\right)}^{2}$$

where $D_{n}$ represents the potential depth, and $a_{n}$ represents the inverse of the width of the potential well. To account for the effect of molecular crowding, such as from polyethylene glycol (PEG), we modify the Morse potential depth locally. The presence of a crowder near a base-pair restricts its thermal fluctuations and stabilizes the hydrogen bond, effectively making it harder to break. We represent this stabilizing effect by scaling the potential depth as $D=\alpha D_{0}$, where α>1 is a phenomenological factor. Although the crowding effect is implemented at a specific site, the nonlinear coupling in the stacking interaction of the PBD model causes this local change to influence the dynamics of neighboring base-pairs as well. It reflects the real physical behavior whereby stiffening one part of the chain influences its surroundings. This approach simplifies the implementation while capturing the local stabilizing effect of crowding. A detailed physical interpretation of this assumption and the role of the scaling factor α is discussed in Section 2.1. The bond strengths of AT and GC pairs are in an approximate ratio of 1.25–1.5 as the GC pairs have three hydrogen bonds, while AT pairs have two. The complete set of parameters used in this study is as follows: $D_{AT}$ = 0.0395 eV, $D_{GC}$ = 0.059 eV, $a_{AT}$ = 4.2 Å^−1^, $a_{GC}$ = 6.3 Å^−1^, ρ = 2.0, κ = 0.03 eV/Å^2^, and *b* = 0.35 Å^−1^. The thermodynamics of the transition is studied by evaluating the partition function, *Z*. For a sequence of *N* base-pairs, the canonical partition function can be defined as follows:

(6) $$Z=\int \prod_{i=1}^{N}\left\{dy_{i}dp_{i}\exp\left(-\beta H\right)\right\}=Z_{p}Z_{c}$$

where $Z_{p}$ corresponds to the momentum part of the partition function and is equal to ${\left(2\pi mk_{B}T\right)}^{N/2}$. The configurational part of the partition function, $Z_{c}$ is defined as

(7) $$Z_{c}=\int_{-\infty }^{\infty }e^{-\frac{\beta V(y_{1})}{2}}dy_{1}\prod_{i=n}^{N-1}dy_{n}e^{-\frac{\beta }{2}\left[V\left(y_{n}\right)+V\left(y_{n+1}\right)+2W\left(y_{n},y_{n+1}\right)\right]}e^{-\frac{\beta V(y_{N})}{2}}dy_{N}$$

We adopt the following method to calculate the partition function for chains with a random sequence of AT and GC pairs and open boundaries. The partition function in the PBD model is divergent; proper cut-offs are required to overcome this issue. In our previous studies, we found that an upper cut-off of 200 Å is sufficient to overcome the divergence issue of the partition function and the lower limit of integration is set as −5.0 Å [47,48,49]. Once we find the proper cut-offs, the task is to discretize the integral in Equation (7). We use the Gaussian quadrature to integrate the equation of partition function numerically. We discretize the configurational space into 900 points. Once we evaluate the partition function, we determine the thermodynamic quantities of interest by evaluating the Helmholtz free energy of the system. The Helmholtz free energy per base-pair is defined as

(8) $$f\left(T\right)=-\frac{1}{2}k_{B}T\ln\left(2\pi mk_{B}T\right)-\frac{k_{B}T}{N}\ln Z_{c}$$

In the thermal ensemble, the specific heat, $C_{v}$, is evaluated by taking the second derivative of the free energy, $C_{v}=-T\left(\partial^{2}f/\partial T^{2}\right)$. We calculate the melting temperature ($T_{m}$) from the peak in the specific heat curve. In the experiments, researchers monitor the fraction of open pairs, ϕ, as a function of temperature using various spectroscopic techniques. To calculate the ϕ, we adopt the method as discussed by Campa et al. [44] and calculate the value of θ (ϕ=1−θ), average fraction of intact pairs as a function of temperature.

## 4. Conclusions

The presence of crowders influences the conformation and stability of DNA molecules and the PBD model has been used to study the effects of crowding on DNA duplexes, demonstrating its versatility in capturing the restricted movement of base-pairs in various crowding locations along DNA. We investigated how the presence of crowders affects the melting of DNA molecules by analyzing the melting profiles of both short and long DNA chains. The melting temperatures of DNA molecules with crowders were calculated and compared to experimental melting profiles. With appropriate modifications to the PBD model for crowded base-pairs, we could identify the likely locations of these crowders, as according with experimental conditions. By systematically evaluating various crowder arrangements, we provided a clear assessment of the impact of crowders on DNA melting profiles. In living organisms, the strength of the crowders surrounding a specific base-pair can significantly differ from those around other base-pairs along the DNA chain. Therefore, we investigated the effect of crowders of different strengths on the melting behavior of heterogeneous DNA molecules of different lengths. The microscopic characteristics of DNA melting vary between short and long DNA chains. The presence of multiple peaks in the specific heat indicates that the DNA melting process is complex and involves distinct probabilities for strand opening. These findings complement non-uniform DNA melting in vivo, driven by crowding heterogeneity. The longer DNA chains exhibit lower melting temperatures than shorter ones in the presence of similar crowders, which indicates that they are more susceptible to melting due to enhanced entropy and cooperative base-pair opening along the extended chain. The melting of short DNA chains is characterized by localized, stepwise unzipping, while long DNA chains exhibit more cooperative, slithering-based melting that resembles a phase transition. This variation plays a crucial role in the dynamics and stability of dsDNA and has potential implications for various fields, including biophysics, biochemistry, and the study of DNA-related diseases. The present study is an attempt to understand the complex stability of DNA molecules in crowded environments. The PBD model effectively accounts for the restricted movement of base-pairs in crowded environments, providing insights into DNA behavior under cellular-like conditions. In conclusion, this study effectively bridges the gap between theory and experiment by offering a modified PBD model that (i) accurately replicates experimental melting profiles and (ii) predicts how DNA stability is influenced by sequence and the arrangement of crowders. Future research will specifically examine how the duration of a crowder’s presence at a specific location along the DNA chain impacts the melting of DNA molecules. 

## Data Availability

Data is contained within the article and Supplementary Materials.

## Supplementary Materials

The following supporting information can be downloaded at https://www.mdpi.com/article/10.3390/ijms26115305/s1.
