# Supplementary material for: Melting Profile of DNA in Crowded Solution: Model-Based Study
Source: Int J Mol Sci. 2025 May 31;26(11):5305. doi: 10.3390/ijms26115305 (PMC12154979; doi:10.3390/ijms26115305)
Supplement: Supplementary file 1 [file ijms-26-05305-s001.zip › ijms-3627208-supplementary.pdf]

Supplementary Materials: Melting Profile of DNA in Crowded Solution: Model-Based Study

Neha Mathur [1], Amar Singh [2,*], Navin Singh [1,]

Supplementary Table S1: Melting temperature (T_m) values for different crowder configurations.

**Crowder distribution**	2,3,7	1,4,7	1,4,8	6,3,4	1,3,5	1,4,6	4,6,7	3,4,5
$Tm(K)$	289.8	290.0	292.0	293.9	296.8	299.5	301.3	298.0
**Crowder distribution**	2,3,6	3,4,8	3,5,6	3,6,8	4,6,8	2,3,4	3,4,7	4,5,6
$Tm(K)$	297.4	297.9	298.3	297.9	300.5	298.7	298.7	299.8
**Crowder distribution**	1,2,3	6,7,8	8,5,4	5,6,7	1,2,6	1,3,7	1,3,8	1,4,5
$Tm(K)$	303.5	303.1	303.4	303.0	303.4	303.0	303.2	303.4
**Crowder distribution**	2,3,8	2,4,7	2,4,8	2,5,3	2,5,6	2,6,8	3,5,8	3,7,8
$Tm(K)$	303.2	304.2	305.2	303.5	305.2	303.5	303.5	304.2
**Crowder distribution**	5,6,8	1,2,4	2,6,7	3,5,7	1,5,8	2,4,5	1,2,8	1,7,8
$Tm(K)$	303.2	304.6	306.8	305.9	307.4	306.4	308.3	309.8
**Crowder distribution**	1,5,7	2,5,7	5,7,8	1,2,5	1,2,7	2,4,6	1,3,6	1,6,8
$Tm(K)$	311.9	314.5	313.0	310.8	310.9	317.8	298.3	303.2
**Crowder distribution**	1,3,4	3,6,7	1,5,6	1,6,7	4,5,7	4,7,8	2,5,8	8,7,2
$Tm(K)$	298.0	299.8	303.0	303.1	303.4	304.2	309.8	313.8

Table S1. Melting temperatures (T_m, in K) for all 56 unique configurations of three crowders on the DNA duplex referred to as chain-A in the main text.

Supplementary Figure S1:

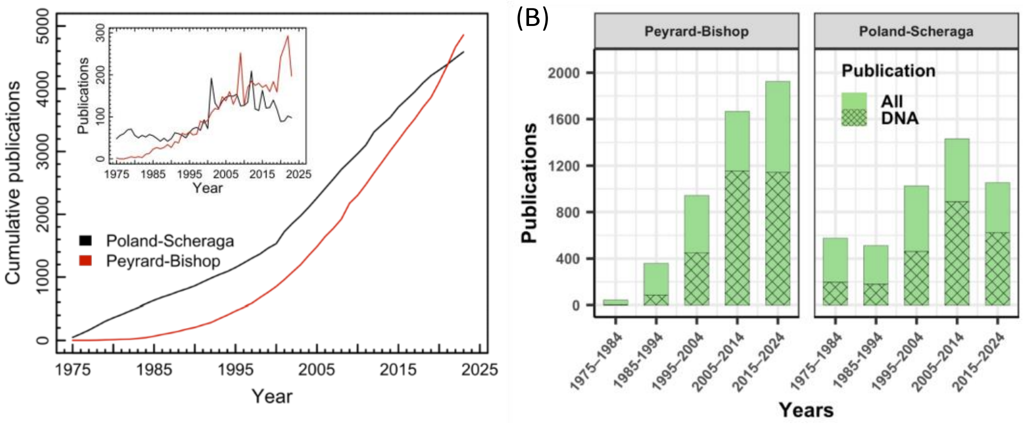

(A)

Figure S1. (A) Cumulative number of publications mentioning the theoretical Poland–Scheraga or Peyrard–Bishop models from 1975 to 2024. Inset: Annual publication counts for the same models over the same time period. (B) Fraction of publications every ten years mentioning DNA. The data were obtained on June 2024 from Digital Science's Dimensions platform, available at https://app.dimensions.ai.

Supplementary Figure S2: Average separation ⟨y⟩ of base-pairs in DNA duplex. 3

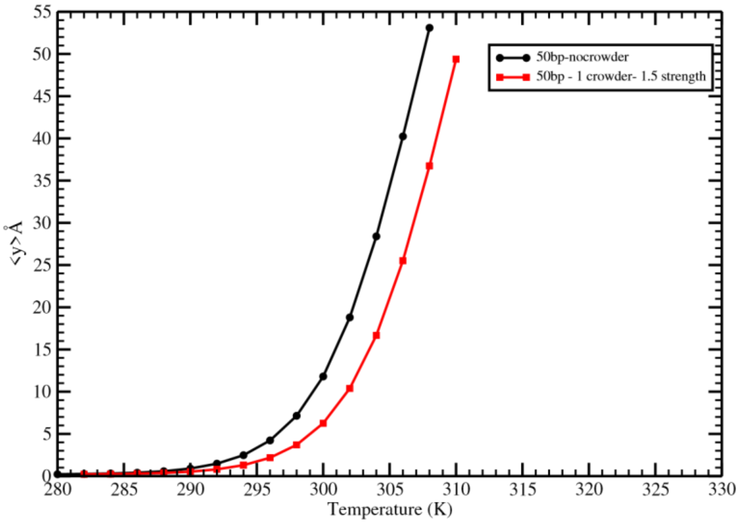

Figure S2. Average base-pair separation ⟨y⟩ in DNA duplex as a function of temperature, T. A divergence in ⟨y⟩ is observed near y_0 = 2.0 Å.

Supplementary Figure S3: DNA melting profiles under different crowding conditions. 4

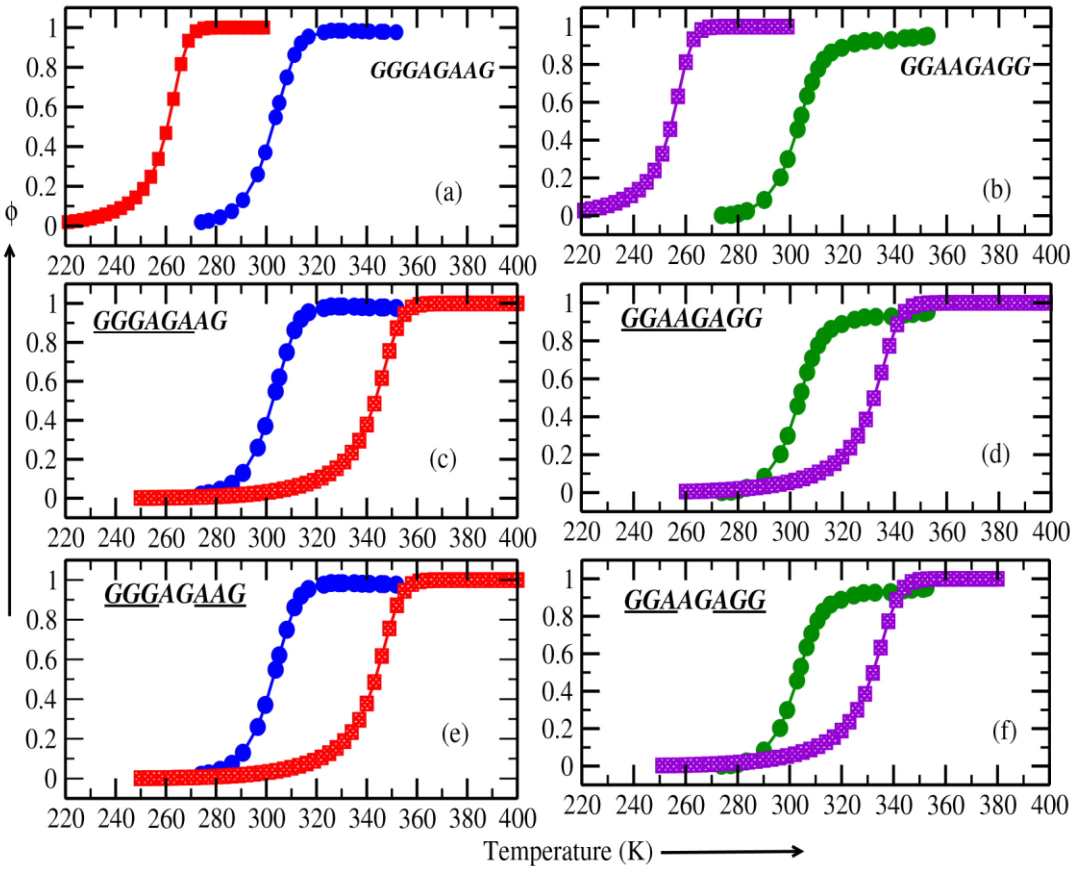

Figure S3. Melting profiles of DNA sequences under different crowding conditions. In the left panels (a, c, e), the **blue curves** represent experimental results for chain-A (GGGAGAAG), while the **red curves** correspond to theoretical predictions using the PBD model. In the right panels (b, d, f), the **green curves** show experimental results for chain-B (GGAAGAGG), and the **purple curves** represent the corresponding PBD model predicted melting curves. (a) and (b): Melting curves for chain-A and chain-B, respectively, in the **absence of crowders**. (c) and (d): Melting curves for the same chains with **six crowders** uniformly placed at positions 1–6, representing a case of **doubled crowding** relative to analysis in main text. (e) and (f): Melting curves with **six crowders placed at asymmetric positions** (1, 2, 3, 6, 7, and 8) to evaluate the sensitivity of melting behavior to crowder distribution.
